# Supplementary material for: Humid heat environment causes anxiety-like disorder via impairing gut microbiota and bile acid metabolism in mice
Source: Nat Commun. 2024 Jul 7;15:5697. doi: 10.1038/s41467-024-49972-w (PMC11228019; doi:10.1038/s41467-024-49972-w)
Supplement: Supplementary file 1 — Supplementary Information [file 41467_2024_49972_MOESM1_ESM.pdf]

Supplementary Figure 1

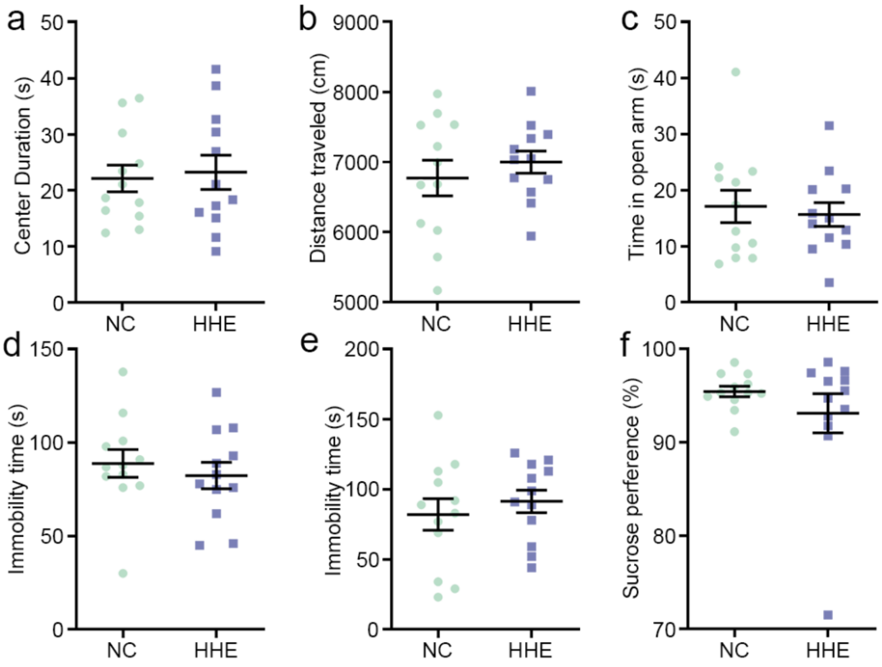

Supplementary Figure 1. Female mice housed in the humid heat environment show no behavioral abnormalities. All female mice were divided into the HHE group and the NC group, housed in the humid heat and the normal conventional environment for 45 days respectively. **a, b** In the open-field test, the central duration and the total moving distance were comparable in two groups. **c** There were no differences of staying in the close arms of the the elevated plus maze in two groups. **d, e** The immobility time in the tail suspension test and the forced swimming was similar in two groups. **f** There were no differences of sucrose preference in two groups. Statistical analysis by two-tailed *t*-test. n=12 mice/group. All data are presented as mean values +/− SEM. Source data are provided as a Source data file.

Supplementary Figure 2

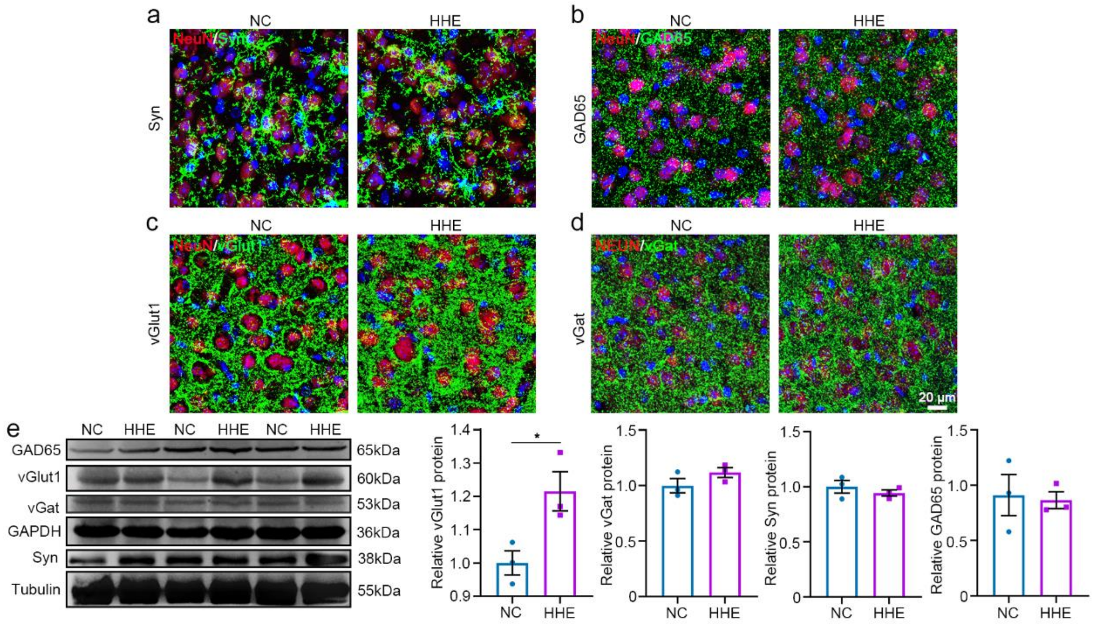

Supplementary Figure 2. Humid heat environment results in the elevation of vGlut1 expression in the cortex. a-d After 45-dyas housing in the controlled environment, brain sections were prepared for anti-NeuN fluorescent staining (red) together with anti-synaptophysin (Syn, green, **a**), anti-GAD65 (green, **b**), anti-vGlut1 (green, **c**), and anti-vGAT (green, **d**). **e** Western blots of cortical samples showed a significant increase of vGlut1 and the comparable levels of vGAT, Syn and GAD65 in the HHE group compared to the NC group. Statistical analysis by two-tailed *t*-test. n=3 mice/group. *, $P<0.05$. GAPDH and tubulin as reference controls. All data are presented as mean values +/− SEM. Source data are provided as a Source data file.

Supplementary Figure 3

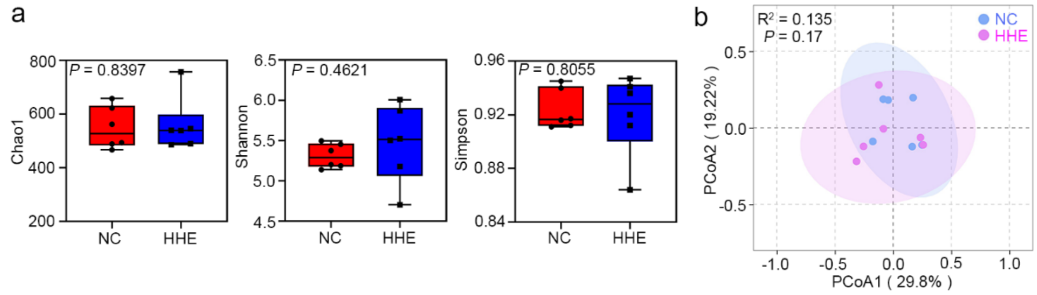

Supplementary Figure 3. Baseline diversity of gut microbiota is similar in the NC and the HEE groups. a, b Before the conditioned housing, Alpha and beta diversity of the baseline microbiota showed no differences in the NC and the HHE groups (two-tailed Mann−Whitney U test and PERMANOVA, respectively). In box plot (a), the lines from top to bottom represent maximum, 3rd quartile, median, 1st quartile, and minimum, while the middle area represents the Interquartile range. All data are presented as mean values +/− SEM. Source data are provided as a Source data file.

Supplementary Figure 4

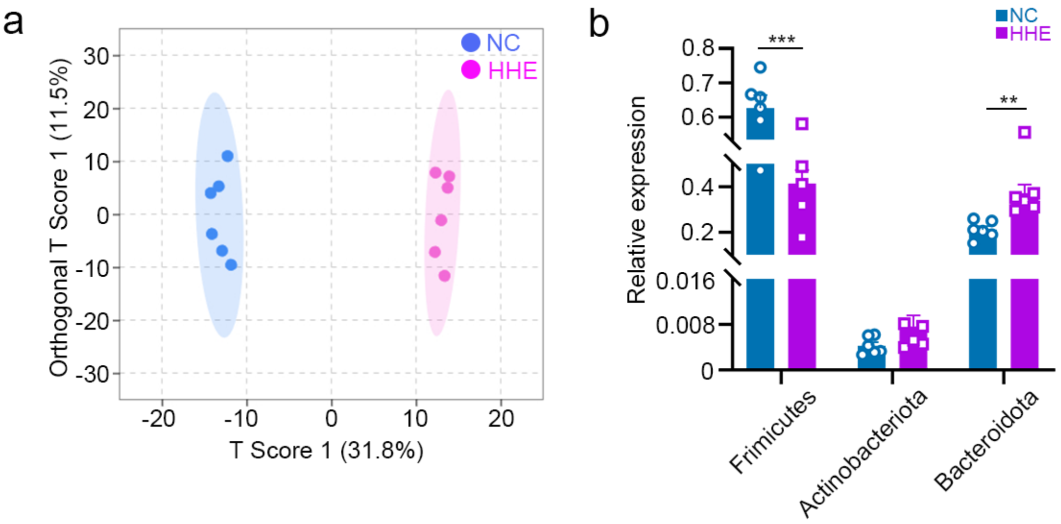

Supplementary Figure 4. Humid heat environment alters serum metabolite composition and phylum levels of the bacterial groups. a The serum metabolic profile was significantly different between the NC and HHE groups identified by the OPLS-DA method. The x-axis captured the variation between the groups, while the y-axis captured the variation within the groups. **b** Statistical graph showed the relative abundances of Firmicutes, Actinobacteria and Bacteroidetes in two groups. Two-way ANOVA, Bonferroni's multiple comparison test. n=6 mice/group. All data are

presented as mean values +/− SEM. **, *P*<0.01; ***, *P*<0.001. Source data are provided as a Source data file.

Supplementary Figure 5

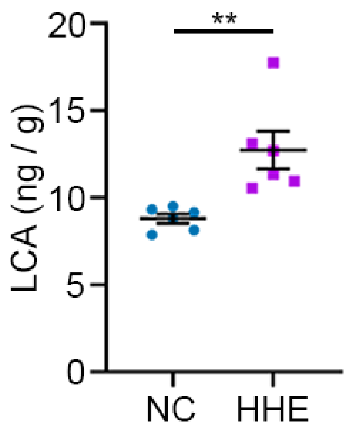

Supplementary Figure 5. Lithocholic acid increased in the brain. LCA concentration measured by targeted mass spectrometry in the brain was significantly increased in the HHE group compared to the NC group. Statistical analysis by two-tailed *t*-test; n=6 mice/group. All data are presented as mean values +/− SEM. **, *P*<0.01. Source data are provided as a Source data file.

Supplementary Figure 6

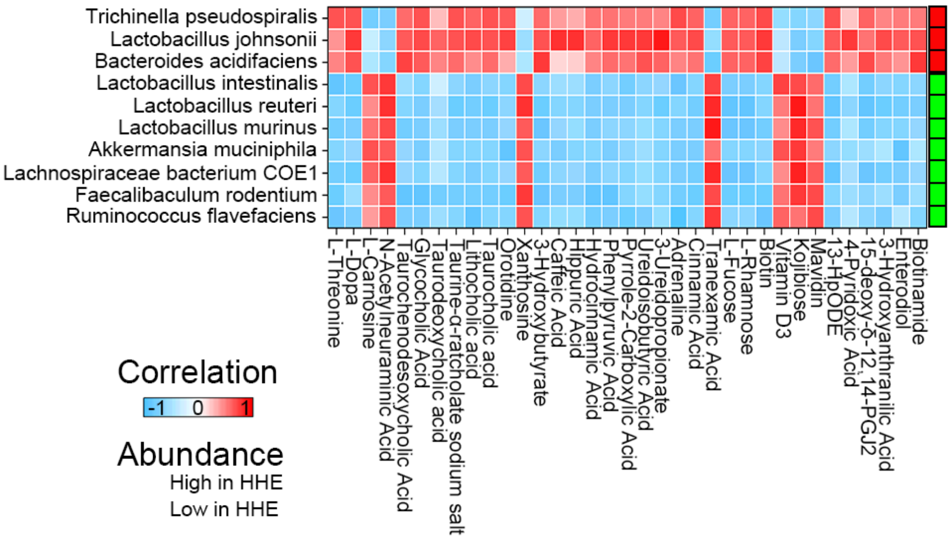

Supplementary Figure 6. Altered abundance of gut microbiota is correlated to expression levels of serum metabolites. Heatmap showed the correlation strength (negative to positive: the color from blue to red) between abundance changes of different gut microbiota and expression alterations of different serum metabolites using partial Spearman correlation. n=6 mice/group.

Supplementary Figure 7

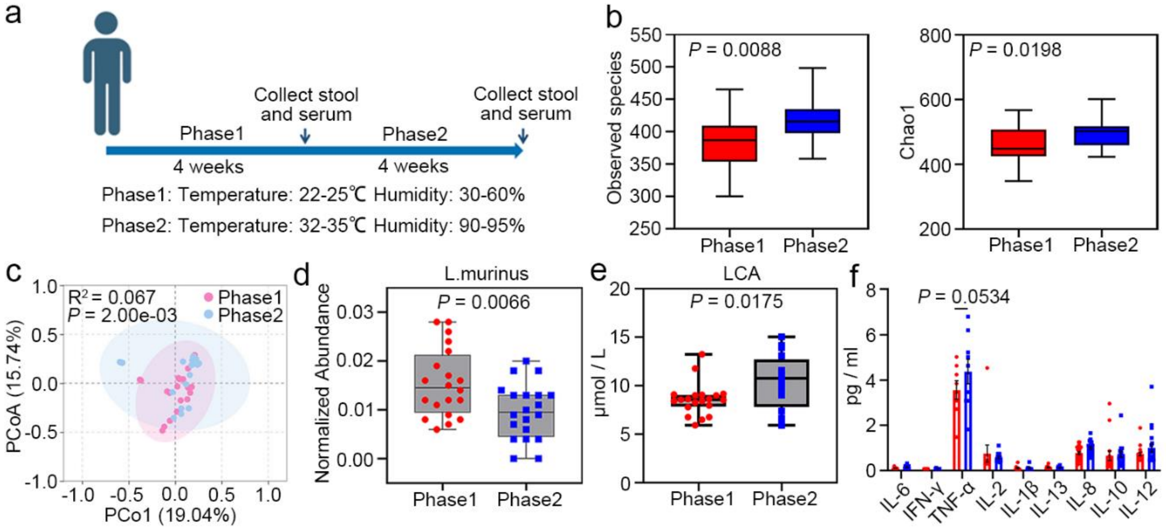

Supplementary Figure 7. Humid heat season impairs gut microbiota and upregulate serum LCA in people. a The schema illustrates the experiment design, in which faecal samples of individual participant were collected at the end of the first and the second phases (about 4 weeks in each phase) during the season transition, defined as phase 1 or phase 2 respectively. **b, c** 16S-RNA sequencing of human faecal samples identified a significant difference of gut microbiota as shown in alpha diversity of observed species and Chao1 analysis and beta diversity of PCoA ratio between phase 1 and phase 2 (a two-tailed Mann–Whitney U test and PERMANOVA, respectively). **d, e** Compared to the phase 1, the abundance of *Lactobacillus murinus* was significantly decreased (P<0.05, normalized by arcsine square root calculation), whereas serum LCA concentration was increased in the phase 2 (P<0.05, Student's *t*-test). **f** Inflammatory cytokines in the serum samples were detected by the Proinflammatory Panel 1 Kits, and there was an increase trend of proinflammatory cytokines TNF-α in the phase 2 compared to phase 1 (P=0.0534, two-way ANOVA and Bonferroni's multiple comparisons test). n=20 subjects. In box plot (b, d, e), the lines from top to bottom represent Maximum, 3rd quartile, Median, 1st quartile, and Minimum, while the middle area represents the Interquartile range. Data are presented as mean values +/− SEM. Illustrations created with BioRender.com. Source data are provided as a Source data file.

Supplementary Figure 8

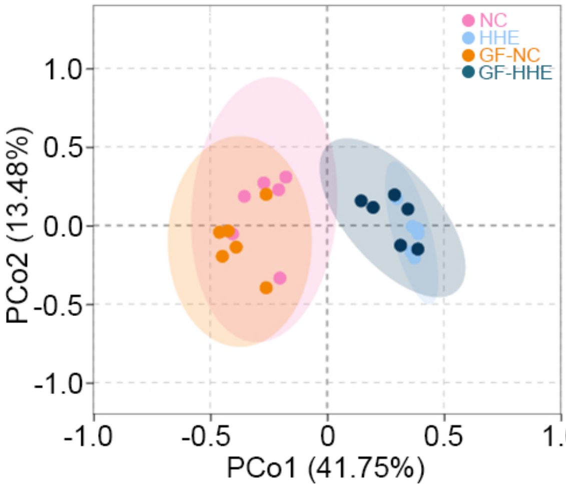

Groups	R^2	*P*
NC vs GF-NC	0.147	0.15
HHE vs GF-HHE	0.086	0.5
NC vs HHE	0.44	0.0027
GF-NC vs GF-HHE	0.45	0.0029

Supplementary Figure 8. Faecal microbiota transplantation recapitulates beta diversity of gut microbiota in GF mice. PERMANOVA analysis disclosed the correlations of the gut microbiota beta diversity among the NC, HHE, GF-NC and GF-HHE groups, showing the similarities in the NC *versus* the GF-NC and the HHE *versus* the GF-HHE, but the significant differences in NC *versus* the HHE and the GF-NC *versus* the GF-HHE. n=6 mice/group.

Supplementary Figure 9

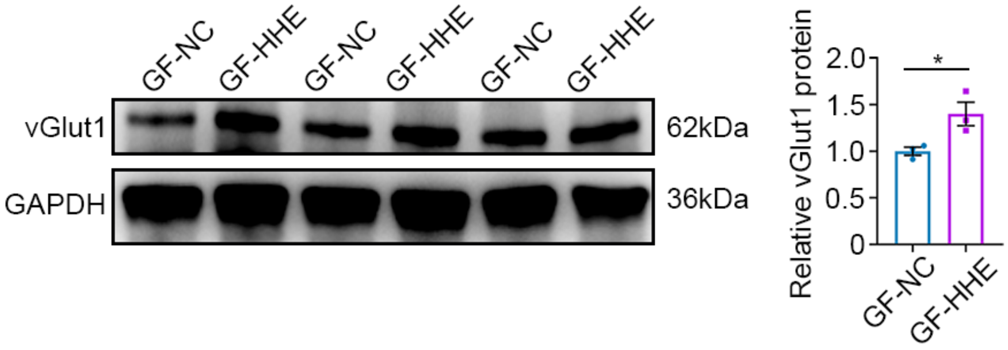

Supplementary Figure 9. Faecal microbiota transplantation upregulates cortical vGlut1 expression in GF mice. Western blots of cortical samples showed a significant increase of vGlut1 expression in the GF-HHE group compared to the GF-NC group. *, P<0.05; Student's t-test; n =3 mice/group. GAPDH as a reference control. All data are presented as mean values +/− SEM. Source data are provided as a Source data file.

Supplementary Figure 10

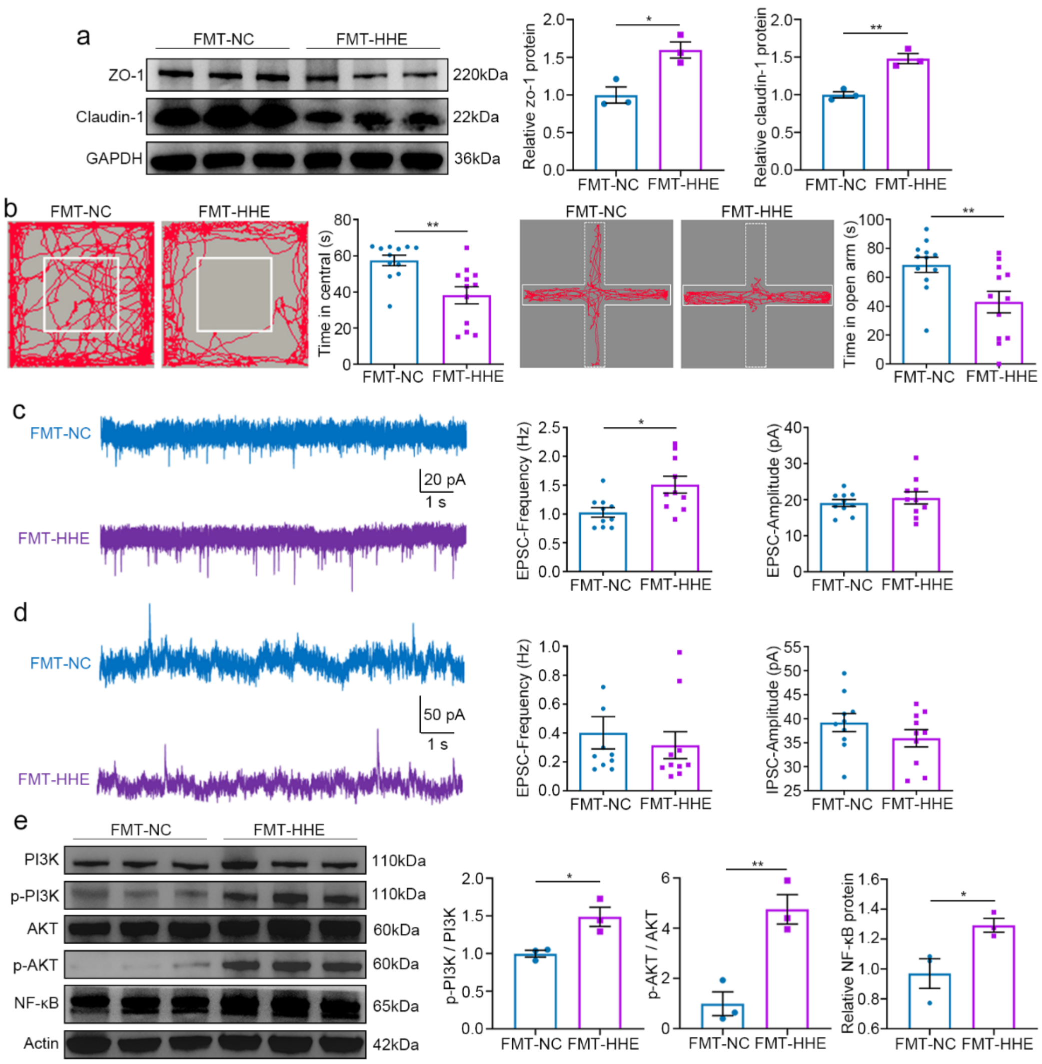

Supplementary Figure 10. Faecal transplantation recapitulates alteration molecular features and anxiety-like behaviors in FMT mice. a In the FMT-HHE group, protein expression of claudin-1 and ZO-1 was significantly decreased as shown in western blots of the colon samples, n=3 mice/group. **b** There was a significant increase of travelling the central zone of the open field and staying close arms of elevated plus maze in the FMT-HHE group compared to the FMT-NC group, n=12 mice/group. **c, d** Mouse acute brain slices were prepared and subjected to electrophysiological recordings of sEPSCs and sIPSCs in pyramidal neurons located in the layer II/III of the mPFC. sEPSC recordings showed a significant decrease of frequency in the FMT-HHE group compared to the FMT-NC group, and no differences of sEPSC amplitude in two groups (**c**). In sIPSC recordings, no differences of frequency and amplitude were identified by the analysis in two groups (**d**). Total 10 neurons in

each recording and 3 mice in each group. **e** Western blots of cortical samples showed a significant decrease of phosphorylated PI3K and AKT1, total NF-κB proteins in the FMT-HHE group compared to the FMT-NC group. *, P<0.05; **, P<0.01; Student's *t*-test; n=3 mice/group. All data are presented as mean values +/− SEM. Source data are provided as a Source data file.

Supplementary Figure 11

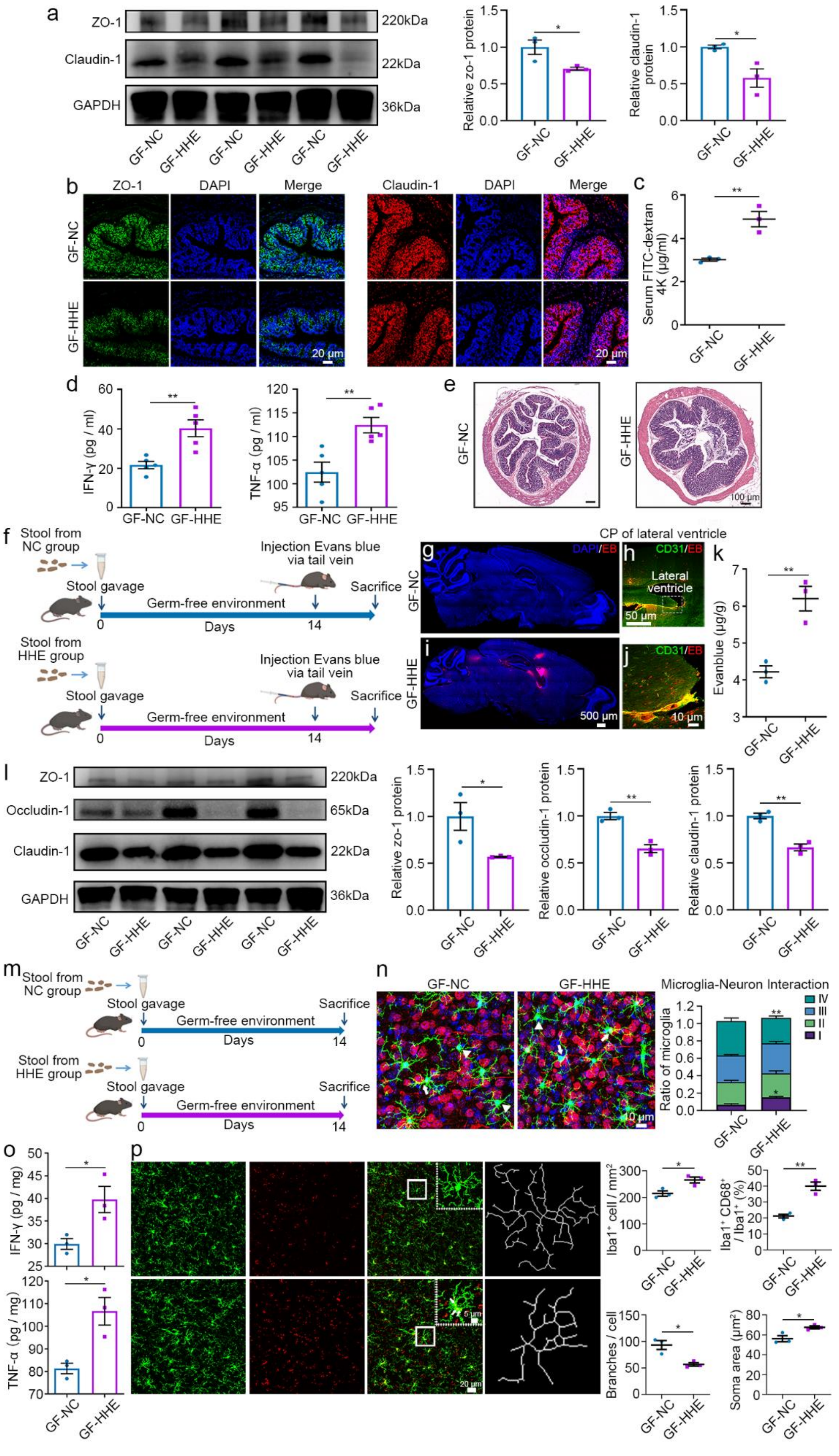

Supplementary Figure 11. Faecal transplantation increases the permeability of gut barrier and BBB and promotes neuroinflammation in GF mice. a, b Claudin-1 and

ZO-1 was as shown in Western blots of the colon samples and immunofluorescence staining of the colon sections compared to the GF-NC group. DAPI (blue) counterstained nuclei. **c** Statistics showed serum FITC-dextran. **d** The inflammatory factors IFN-γ and TNF-α in the brains detected by ELISA. **e** H.E. stain of colon tissue. *, *P*<0.05; **, *P*<0.01; Statistical analysis by two-tailed *t*-test; n=3 mice/group in **a-c, e**; n=5 mice/group in **d**. **f** EB tracing and the experimental outflow was illustrated in the diagram. **i, g** Abundant EB signal (red) was visible in the GF-HHE group (**i**), but rare in the GF-NC group (**g**). **h, j** Confocal images showed that EB-positive signal. **k** Statistics showed the concentration of EB in the brain. **l** Representative image and statistical analysis of the claudin-1, occludin-1 and zo-1 protein levels. Statistical analysis by two-tailed *t*-test. *, *P*<0.05; **, *P*<0.01; n=3 mice/group. **m** Schematic diagram showing the experimental procedures. **n** In cortical sections immunostained for Iba1 (green) and NeuN (red), white arrows point to I type and white triangles point to IV type. Statistical analysis by two-tailed *t*-test. n=3 mice/group. **o** Representative images of microglia and statistics showed Iba1-positive cell density, the percentage of double positive cells, average microglial soma area and average microglia branch number. n=3 mice/group. Statistical analysis by two-tailed *t*-test, n=3 mice/group. All data are presented as mean values +/− SEM. *, *P*<0.05; **, *P*<0.01. Illustrations created with BioRender.com. Source data are provided as a Source data file.

Supplementary Figure 12

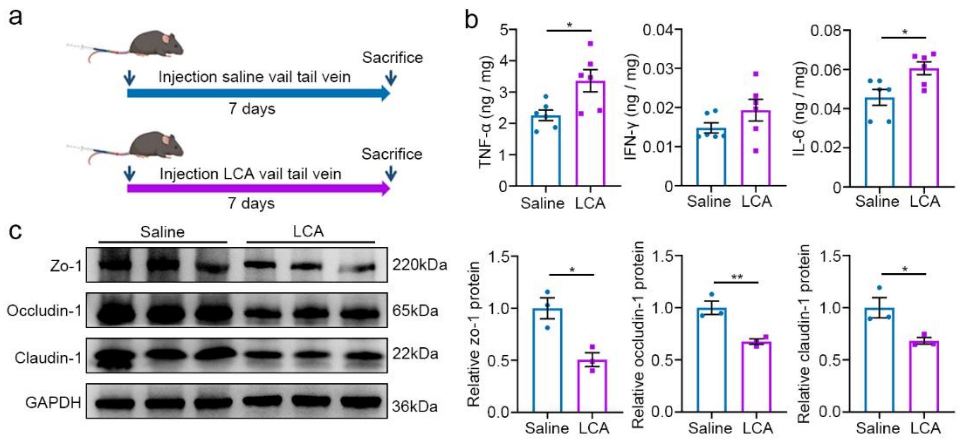

Supplementary Figure 12. Increased lithocholic acid induced the permeability of BBB and promotes neuroinflammation in mice. a The level of inflammation factors

and BBB impermeability was studied by injection LCA vail tail vein and the experimental outflow was illustrated in the diagram. **b** The inflammatory factors TNF-α and IL-6 in the brains of LCA group detected by ELISA was a significant increase compared to the Saline group. Statistical analysis by two-tailed *t*-test; *, *P*<0.05; **, *P*<0.01; n=6 mice/group. **c** Protein levels of claudin-1, occludin-1 and zo-1 in the cortical samples were significantly decreased as shown in Western blots in the LCA group (Statistical analysis by two-tailed *t*-test. *, *P*<0.05; **, *P*<0.01; n=3 mice/group). All data are presented as mean values +/− SEM. Illustrations created with BioRender.com. Source data are provided as a Source data file.

Supplementary Figure 13

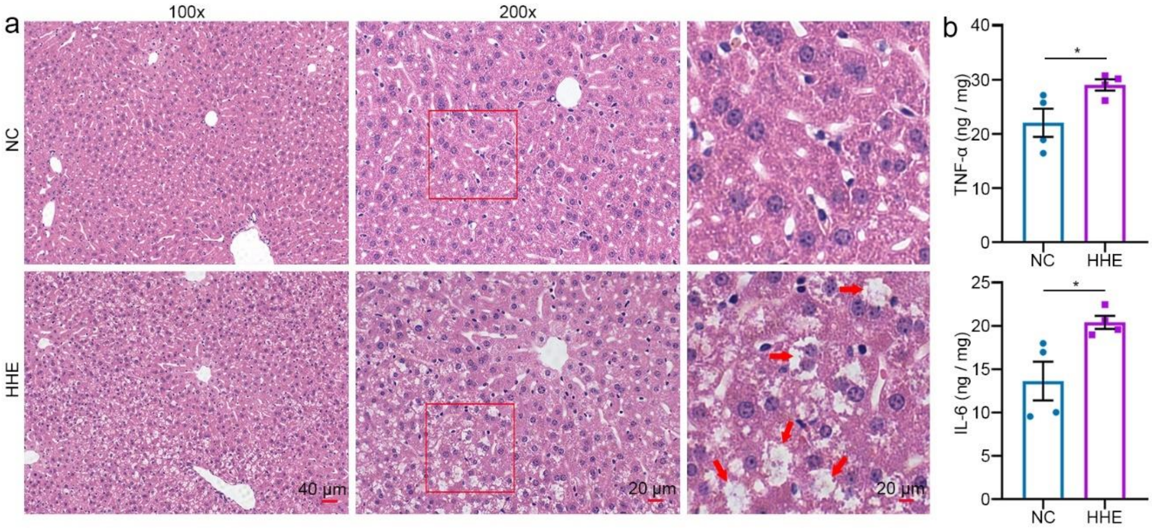

Supplementary Figure. 13. HHE induces mouse live damage and inflammatory cytokine secretion. a H&E staining showed lipid deposition (red arrows) in the HHE group but rarely in the NC group. **b** ELISA of liver samples showed a significant increase of TNF-α and IL-6 in the HHE group compared to the NC group. Statistical analysis by two-tailed *t*-test; *, *P*<0.05; n=4 mice/group. All data are presented as mean values +/− SEM. Source data are provided as a Source data file.

Supplementary Figure 14

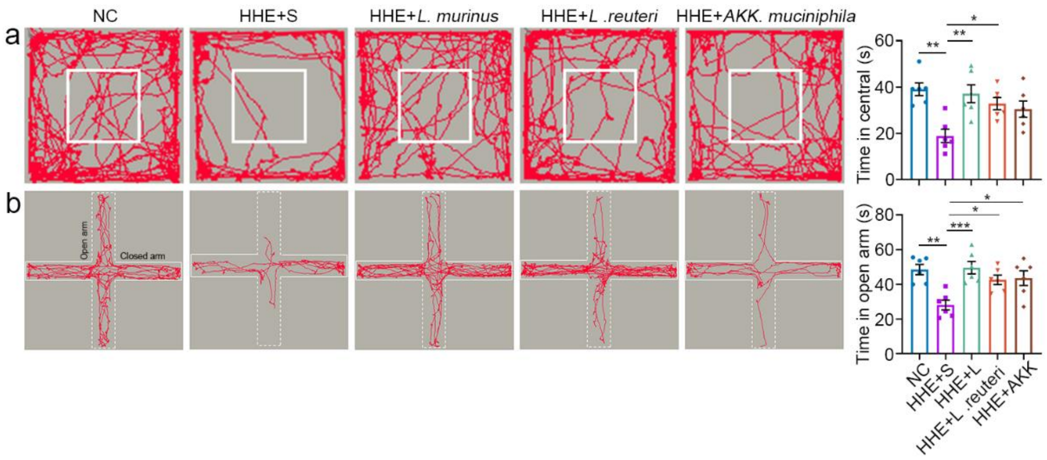

Supplementary Figure 14. ***Lactobacillus murinus*** **have the better improvement for HHE-induced anxiety disorder compared to** ***Lactobacillus reuteri*** **and** ***Akkermansia muciniphila.*** **a, b** There was a significant increase of travelling the central zone of the open field and staying open arms of elevated plus maze in the both HHE+L group and HHE+L. reuteri group compared to the HHE+S group. HHE+Akk group spent more time stay open arms of elevated plus maze compared to the HHE+S group. n=6 mice/group; Statistical analysis by one-way ANOVA with Bonferroni's post hoc test. *, P<0.05; **, P<0.01; ***, P<0.001; All data are presented as mean values +/− SEM. Source data are provided as a Source data file.

Supplementary Figure 15

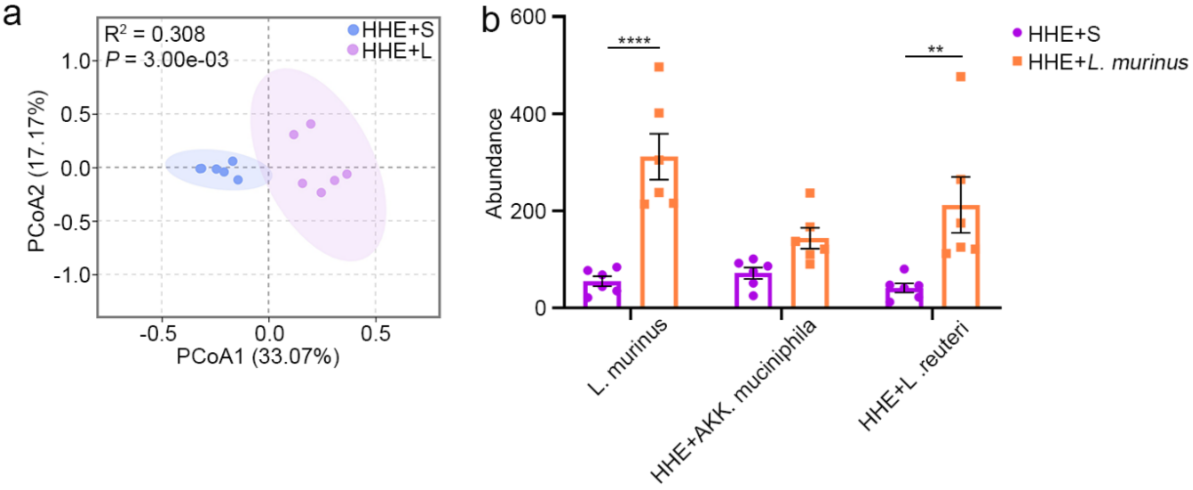

Supplementary Figure 15. a In two groups, unweighted UniFrac distance-based analysis showed the significant differences of microbial composition in the PCoA (beta diversity). **b** The abundance of *Lactobacillus murinus*, *Akkermansia muciniphila*

and *Lactobacillus reuteri* changes induced by *Lactobacillus murinus* treatment. two-way ANOVA with Bonferroni's post hoc test. **, $P<0.01$; ****, $P<0.0001$. n= 6mice/group. All data are presented as mean values +/− SEM. Source data are provided as a Source data file.

Supplementary Figure 16

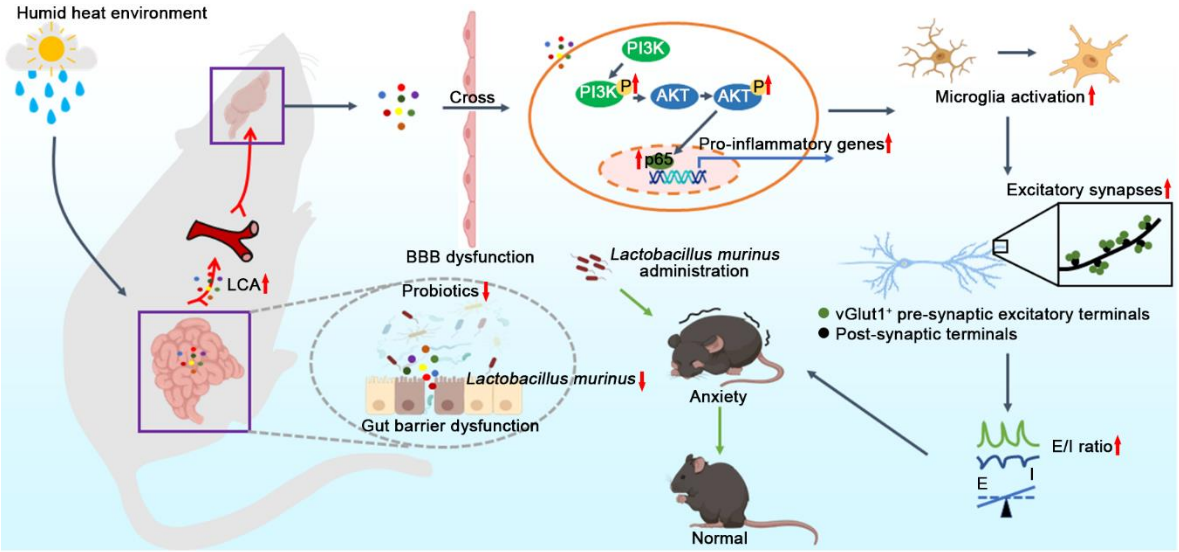

Supplementary Figure 16. Humid heat environment promotes anxiety disorder by inducing gut microbiota dysbiosis and changed metabolites.

Humid heat environment induced gut microbiota dysbiosis (eg. induced Lactobacillus murinus) and increased gut microbiota-related metabolites in serum (eg. induced lithocholic acid (LCA)), which enhanced neuroinflammation was demonstrated by the elevated expression of proinflammatory gene in the cortex, the activated PI3K/AKT/NF-κB signalling and microglial response in the cortex. The activation microglia affected the cortical neurons which displayed increased AMPA receptor mediated spontaneous excitatory currents associated with an increase in the number of excitatory pre-synaptic terminals in the cortex and finally disrupt E/I balanced caused anxiety behavior in mice. (Created with BioRender.com.)

Supplementary Table 1

REAGENT or RESOURCE	SOURCE	IDENTIFIER
Antibodies		
Anti-Synaptophysin (For IHC-F or WB)	Abcam	ab8049, 1:100 for IHC-F, 1:1000 for WB
Anti-GAD65 (For IHC-F or WB)	Abcam	ab239372, 1:100 for IHC-F, 1:1000 for WB
Anti-VGluT1 (For IHC-F or WB)	Abcam	ab227805, 1:100 for IHC-F, 1:1000 for WB
Anti-VGAT (For IHC-F)	Abcam	ab308062, 1:50
Anti-VGAT (For WB)	Thermo Fisher	MA5-47111, 1:1000
Anti-ZO1 (For IHC-P)	Abcam	ab221547, 1:100
Anti-ZO1 (For WB)	Thermo Fisher	33-9100, 1:250
Anti-Claudin1 (For IHC-P)	Abcam	ab211737, 1:1000
Anti-Claudin1 (For WB)	Cell Signalling Technology	13255S, 1:1000
Anti-Occludin (For WB)	Cell Signalling Technology	91131S, 1:1000
Anti-Iba-1 (For IHC-F)	Wako	019-19741, 1:1000
Anti-NeuN (For IHC-F)	Abcam	ab279296, 1:1000
Anti-(medium) Neurofilament (Clone NF-09)	Acris Antibodies GmbH (now OriGene)	SM3068P, 1:100
Anti-CD68 (For IHC-F)	Thermo Fisher	14-0681-82, 1:200
Anti-PI3K (For WB)	Cell Signalling Technology	4257S, 1:1000
Anti-p-PI3K (For WB)	Cell Signalling Technology	17366S, 1:1000
Anti-AKT (For WB)	Cell Signalling Technology	4691S, 1:1000
Anti-p-AKT (For WB)	Cell Signalling Technology	4060S, 1:1000
Anti-rabbit IgG (HRP)	R&D System	HAF008, 1:1000
Anti-mouse IgG (HRP)	R&D System	HAF007, 1:1000
Alexa Fluor 488 goat anti-rabbit IgG	Thermo Fisher	A21206, 1:1000
Alexa Fluor 488 goat anti-mouse IgG	Thermo Fisher	A21202, 1:1000
Alexa Fluor 594 goat anti-rabbit IgG	Thermo Fisher	A21207, 1:1000
Alexa Fluor 594 goat anti-mouse IgG	Thermo Fisher	A21203, 1:1000
Alexa Fluor 488 goat anti-rat IgG	Thermo Fisher	A21208, 1:1000
Alexa Fluor 594 goat anti-rat IgG	Thermo Fisher	A21209, 1:1000
Bacterial		
Lactobacillus murinus	BNCC	BNCC194688
Lactobacillus reuteri	BNCC	BNCC192190
Akkermansia muciniphila	BNCC	BNCC341917
Biological samples		
Human fecal and serum samples	The First Affiliated Hospital of Jinan University	N/A
Chemicals, peptides, and recombinant proteins		
Bicuculline	Tocris	2503/10

NBQX	Tocris	0373/10
D-AP5	Tocris	0106/1
DAPI	Thermo Fisher	D3571
TriZol	Thermo Fisher	15596026
TB Green® Premix Ex Taq™ II	Takara	RR047A
RIPA Buffer	Thermo Fisher	89901
Protease and phosphatase inhibitors cocktail	Thermo Fisher	78440
Western HRP Substrate	Millipore	WBLUF0500
FITC-dextran	Sigma	FD4
Evans blue	Tokyo Chemical Industry	E0197
Lithocholic acid	MCE	HY-B0172
PVDF membranes	Millipore	IPVH85R
Isoflurane	RWD	R510-22-10
Methanol	Aladdin	M116118
Triton X-100	Sangon Biotech	A110694-0100
Optimal Cutting temperature (O.C.T)	Sakura	4583
Critical commercial assays		
PrimeScript™ RT Reagent Kit with gDNA Eraser	Takara	RR047A
QIAamp DNA Stool Mini Kit	Qiagen	51504
Bio-Plex Pro™ Reagent Kit	Bio-Rad	M60009RDPD
Proinflammatory Panel 1 (human) Kit	MSD	K15049D
TNF-α ELISA kit	Beijing 4A Biotech	CME0004
IFN-γ ELISA kit	Beijing 4A Biotech	CME0003
IL-6 ELISA kit	Beijing 4A Biotech	CME0006
Pierce BCA Protein assay kit	Thermo Fisher	23225
RNA 6000 Nano LabChip Kit	Agilent	5067-1511
Deposited data		
Raw RNA seq data	Genome Sequence Archive	PRJCA026649
Resources for the 16SrDNA analysis	Genome Sequence Archive	PRJCA026637, PRJCA025700, PRJCA026953
Targeted Metabolomic data	MetaboLights	MTBLS10320, MTBLS10326
Experimental Models: Organisms/Strains		
C57BL/6J mice	SPF (Guangzhou) Biotechnology	N/A
Germ-free mice on C57BL/6J background	Germ-free Clean Animal Facility, The First Affiliated Hospital of Sun Yat -sen University	N/A
Oligonucleotides		

Specific primers for Firmicutes	This study	N/A
Firmicutes forward primer (5'-ATGTGGTTTAATTCGAAGCA-3')		N/A
Firmicutes reverse primer (5'-AGCTGACGACAACCATGCAC -3')		N/A
Specific primers for Actinobacteria	This study	N/A
Actinobacteria forward primer (5'-CGCGGCCTATCAGCTTGTTG -3')		N/A
Actinobacteria reverse primer (5'-CCGTACTCCCCAGGCGGGG -3')		N/A
Specific primers for Bacteroidetes	This study	N/A
Bacteroidetes forward primer (5'-GGARCATGTGGTTTAATTCGATGAT -3')		N/A
Bacteroidetes reverse primer (5'-AGCTGACGACAACCATGCAG -3')		N/A
Universal primer 16S	This study	N/A
16S forward primer (5'-ACTCCTACGGGAGGCAGCAGT -3')		N/A
16S reverse primer (5'-ATTACCGCGGCTGCTGGC -3')		N/A
Specific primers for Pik3r1	This study	N/A
Pik3r1 forward primer (5'-CAAACCACCCAAGCCCACTACT -3')		N/A
Pik3r1 reverse primer (5'-CCATCAGCAGTGTCTCGGAGTT -3')		N/A
Specific primers for AKT1	This study	N/A
AKT1 forward primer (5'-GGACTACTTGCACTCCGAGAAG -3')		N/A
AKT1 reverse primer (5'-CATAGTGGCACCGTCCTTGATC -3')		N/A
Specific primers for Nfkb1	This study	N/A
Nfkb1 forward primer (5'-GCTGCCAAAGAAGGACACGACA -3')		N/A
Nfkb1 reverse primer (5'-GGCAGGCTATTGCTCATCACAG -3')		N/A
Specific primers for Cxcr2	This study	N/A
Cxcr2 forward primer (5'-CTCTATTCTGCCAGATGCTGTCC -3')		N/A
Cxcr2 reverse primer (5'-ACAAGGCTCAGCAGAGTCACCA -3')		N/A
Specific primers for Cxcl1	This study	N/A
Cxcr2 forward primer (5'-		N/A

TCCAGAGCTTGAAGGTGTTGCC -3’)		
Cxcr2 reverse primer (5’-AACCAAGGGAGCTTCAGGGTCA -3’)		N/A
Specific primers for GAPDH	This study	N/A
GAPDH forward primer (5’-CATCACTGCCACCCAGAAGACTG-3’)		N/A
GAPDH reverse primer (5’-ATGCCAGTGAGCTTCCCGTTCAG -3’)		N/A
Software and algorithms		
GraphPad Prism 8.0	GraphPad Software	https://www.graphpad.com/
SPSS 20.0	IBM	https://www.ibm.com/spss
ImageJ	National Institutes of Health (NIH), USA	https://imagej.nih.gov/ij/download.html
ZEN	ZEISS	https://www.zeiss.com/microscopy/int/products/microscope-software/zen.html
Origin	OriginLab	https://www.originlab.com/
Cytoscape	N/A	https://cytoscape.org/index.html
