# Supplementary material for: Humid heat environment causes anxiety-like disorder via impairing gut microbiota and bile acid metabolism in mice
Source: Nat Commun. 2024 Jul 7;15:5697. doi: 10.1038/s41467-024-49972-w (PMC11228019; doi:10.1038/s41467-024-49972-w)
Supplement: Supplementary file 3 — Description of Additional Supplementary Files [file 41467_2024_49972_MOESM3_ESM.docx]

**Description of Supplementary Files**

File Name: Supplementary Data 1

Description: List of altered microbial products and canonical metabolites (n=35 compounds). This is for the in-house spectral library established and used in this work.

File Name: Supplementary software

Description: Installation and operation guide for Cytoscape 3.10.2.
