# Supplementary material for: Humid heat environment causes anxiety-like disorder via impairing gut microbiota and bile acid metabolism in mice
Source: Nat Commun. 2024 Jul 7;15:5697. doi: 10.1038/s41467-024-49972-w (PMC11228019; doi:10.1038/s41467-024-49972-w)
Supplement: Supplementary file 6 — reporting-summary [file 41467_2024_49972_MOESM6_ESM.pdf]

nature portfolio

Corresponding author(s): Libing Zhou, Xiaoyin Chen

Last updated by author(s): May 31, 2024

Reporting Summary

Nature Portfolio wishes to improve the reproducibility of the work that we publish. This form provides structure for consistency and transparency in reporting. For further information on Nature Portfolio policies, see our Editorial Policies and the Editorial Policy Checklist.

Statistics

For all statistical analyses, confirm that the following items are present in the figure legend, table legend, main text, or Methods section.

n/a	Confirmed	
☐	☒	The exact sample size (*n*) for each experimental group/condition, given as a discrete number and unit of measurement
☐	☒	A statement on whether measurements were taken from distinct samples or whether the same sample was measured repeatedly
☐	☒	The statistical test(s) used AND whether they are one- or two-sided<br>*Only common tests should be described solely by name; describe more complex techniques in the Methods section.*
☒	☐	A description of all covariates tested
☐	☒	A description of any assumptions or corrections, such as tests of normality and adjustment for multiple comparisons
☐	☒	A full description of the statistical parameters including central tendency (e.g. means) or other basic estimates (e.g. regression coefficient) AND variation (e.g. standard deviation) or associated estimates of uncertainty (e.g. confidence intervals)
☐	☒	For null hypothesis testing, the test statistic (e.g. *F*, *t*, *r*) with confidence intervals, effect sizes, degrees of freedom and *P* value noted<br>*Give P values as exact values whenever suitable.*
☒	☐	For Bayesian analysis, information on the choice of priors and Markov chain Monte Carlo settings
☒	☐	For hierarchical and complex designs, identification of the appropriate level for tests and full reporting of outcomes
☐	☒	Estimates of effect sizes (e.g. Cohen's *d*, Pearson's *r*), indicating how they were calculated

Our web collection on statistics for biologists contains articles on many of the points above.

Software and code

Policy information about availability of computer code

Data collection	Noted in Methods section.
Data analysis	Image J 1.51J8, SPSS 12.0, Prism-GraphPad 8.0, CurveExpert 1.4, pClamp11.2.

For manuscripts utilizing custom algorithms or software that are central to the research but not yet described in published literature, software must be made available to editors and reviewers. We strongly encourage code deposition in a community repository (e.g. GitHub). See the Nature Portfolio guidelines for submitting code & software for further information.

Data

Policy information about availability of data

All manuscripts must include a data availability statement. This statement should provide the following information, where applicable:

- Accession codes, unique identifiers, or web links for publicly available datasets
- A description of any restrictions on data availability
- For clinical datasets or third party data, please ensure that the statement adheres to our policy

The authors declare that all data supporting the results of this study are available within the paper and its Supplementary Information. All sequencing data generated in this study is deposited at the Sourceforge: https://sourceforge.net/projects/nchhe/. Raw sequence data are also deposited at the Genome Sequence Archive (GSA) under accession number PRJCA026649. 16S rDNA amplicon sequencing and targeted metabolomic generated in this study have been deposited at: http://doi.org/10.6084/m9.figshare.24431887. Raw 16S rDNA sequence data are also deposited at the GSA under accession number PRJCA026637 and PRJCA025700. The relevant metabolomics raw data generated for this study have been deposited in the MetaboLights (accession IDs: MTBLS10320 and

MTBLS10326). Source data are provided with this paper.

Research involving human participants, their data, or biological material

Policy information about studies with human participants or human data. See also policy information about sex, gender (identity/presentation), and sexual orientation and race, ethnicity and racism.

Reporting on sex and gender	In this study, 20 males were included in the group. To be consistent with animal studies, only male human subjects were grouped for analysis.
Reporting on race, ethnicity, or other socially relevant groupings	All participants are the Han nationality.
Population characteristics	The demographic information of human cohort was listed in Source data. In brief, the group had an average age of 25.65 years and live in a campus located in southern Guangdong Province.
Recruitment	20-45 years old males; no history of smoking or stopping smoking more than 1 year; no incidence of diarrhea in the last 1 month and with regular dietary habits (no overeating or eating a large amount of spicy and stimulating food within 1 month); no use of antibacterial drugs in the last 1 month (Which may confound the gut microbiota). To be consistent with experimental animals, only man were recruited in the experiments described in this manuscript.
Ethics oversight	Ethical approval for this study was obtained from the Research Ethics Board of Research Ethics Committee of the First Affiliated Hospital of Jinan University (Guangzhou, China, JNUKY-2022-012), and all participants signed the agreement.

Note that full information on the approval of the study protocol must also be provided in the manuscript.

Field-specific reporting

Please select the one below that is the best fit for your research. If you are not sure, read the appropriate sections before making your selection.

- [x] Life sciences
- [ ] Behavioural & social sciences
- [ ] Ecological, evolutionary & environmental sciences

For a reference copy of the document with all sections, see nature.com/documents/nr-reporting-summary-flat.pdf

Life sciences study design

All studies must disclose on these points even when the disclosure is negative.

Sample size	The sample size was determined based on literatures from the same field (e.g. Nature 2021:589, 426-430), and complied with animal welfare requirement and animal experimental ethical code.
Data exclusions	According to the instructions of the kit, the cytokines in the human serum concentration <0.08 μg/ml are invalid data,so exclusion criteria if measured concentration＜0.08μg/ml.
Replication	All experiments were performed on multiple animals from different litters. The exact N number has been stated in the figure legend. For qPCR study or ELISA, triplicated biological samples were performed for each animal, and the averaged value was displayed.
Randomization	Mice were randomly assigned into each group before the initiation of experiments.
Blinding	All behavioral tests were performed by persons who were blinded to the group information.

Reporting for specific materials, systems and methods

We require information from authors about some types of materials, experimental systems and methods used in many studies. Here, indicate whether each material, system or method listed is relevant to your study. If you are not sure if a list item applies to your research, read the appropriate section before selecting a response.

Materials & experimental systems

n/a	Involved in the study	
[ ]	[x]	Antibodies
[x]	[ ]	Eukaryotic cell lines
[x]	[ ]	Palaeontology and archaeology
[ ]	[x]	Animals and other organisms
[x]	[ ]	Clinical data
[x]	[ ]	Dual use research of concern
[x]	[ ]	Plants

Methods

n/a	Involved in the study	
[x]	[ ]	ChIP-seq
[x]	[ ]	Flow cytometry
[x]	[ ]	MRI-based neuroimaging

Antibodies

Antibodies used	See details from supplement data: Key Resources Table.
Validation	All antibodies used were commercially availbale and previously validated for the species/specificty (see citations provided on manufactorers website). Appropriate positive and negative controls were performed on each tissue type analyzed. Controls utilizing only primary or only secondary antibodies were performed to test for false positive signal.

Animals and other research organisms

Policy information about studies involving animals; ARRIVE guidelines recommended for reporting animal research, and Sex and Gender in Research

Laboratory animals	C57BL/6 mice (7 weeks old) were purchased from the Laboratory Animal Center of Guangdong Province (Guangzhou, China), and assigned into two groups using a computer based random order generator: housing in a normal conventional environment (22-24°C and 45-55% humidity; the NC group) and in a humid heat environment (31-33°C and 91-95% humidity; the HHE group) (12 mice per group, consisting of four cages with 3 mice per cage). GF mice (7 weeks old, C57BL/6 background) were purchased from the Department of Laboratory Animal Science, the First Affiliated Hospital of Sun Yat-sen University(Guangzhou, China), and housed in the GF environment (12 germ-free mice per group, consisting of four cages with 3 mice per cage). For faecal transplantation, stools were collected from mice in the HHE and NC groups and dissolved in 0.01 M phosphate-buffered saline (PBS) at the final concentration of 0.7g/mL, and the stool (200 µL/day) was transplanted to GF mice through oral gavage for 14 consecutive days. All animals were kept under a 12-hour light/dark cycle with ad libitum access to food and water.
Wild animals	No wild animals were used in this study.
Reporting on sex	Only male mice were used in the experiments described in this manuscript. Except for the behaviour test animals where both males and females were used.
Field-collected samples	No field-collected samples were used in this study.
Ethics oversight	All animal experimental protocols have been pre-approved by the Ethics Committee of Experimental Animals of Jinan University in accordance with Institutional Animal Care and Use Committee guidelines for animal research. (Approval code: IACUC-20210528-14). GF animal experimental protocols have been pre-approved by the Ethics Committee of Experimental Animals of IEC for Clinical Research and Animal Trials of the First Affiliated Hospital of Sun Yat-sen University with Institutional Animal Care and Use Committee guidelines for animal research (Approval code: [2023]152).

Note that full information on the approval of the study protocol must also be provided in the manuscript.

Plants

Seed stocks	N/A
Novel plant genotypes	N/A
Authentication	N/A
